# Reprograming yeast for anti-cancer vinblastine synthesis

**DOI:** 10.1093/lifemeta/loac024

**Published:** 2022-09-22

**Authors:** Tian Ma, Zixin Deng

**Affiliations:** CAS Key Laboratory of Quantitative Engineering Biology, Shenzhen Institute of Synthetic Biology, Shenzhen Institute of Advanced Technology, Chinese Academy of Sciences, Shenzhen, Guangdong 518055, China; State Key Laboratory of Microbial Metabolism, Joint International Research Laboratory of Metabolic & Developmental Sciences, and School of Life Sciences and Biotechnology, Shanghai Jiao Tong University, Shanghai 200030, China; Key Laboratory of Combinatorial Biosynthesis and Drug Discovery, Ministry of Education, Zhongnan Hospital, and School of Pharmaceutical Sciences, Wuhan University, Wuhan, Hubei 430072, China

**In a recent study published in *Nature*, Zhang *et al*. employed an innovative approach by reprogramming and engineering yeast strain for combined biosynthesis of vindoline and catharanthine, followed by an additional *in vitro* chemical step for the successful synthesis of the anti-cancer vinblastine.**

Development and utilization of plant resources have played indispensable roles in the treatment of human diseases, which had been an amazing focus of biomedical research for decades. Monoterpene indole alkaloids (MIAs), the plant secondary metabolites that mainly exist in *Gentianales*, had been a popular family of the medicinal drug research because of their remarkable structural diversities and biological activities. So far, about 3000 MIAs have been reported and dozens of them have been used as clinical drugs. Among them, the widely used anticancer drug vinblastine isolated from the leaves of Madagascar periwinkle plant (*Catharanthus roseus*) [1] was found to inhibit the division of cancer cells, and thus was used along with other chemotherapy agents to treat multiple types of cancer, including lymphomas, testicular cancer, ovarian cancer, breast cancer, etc. Nowadays, the World Health Organization has listed vinblastine as an essential medicine [2]. However, the extraction and purification of vinblastine from periwinkle is difficult and inefficient, as it requires >2000 kg of dried leaves to produce just 1 g of vinblastine [3]. Its highly complex structure also makes it difficult to be chemically synthesized. Like all plant-derived natural products, the resource supply of vinblastine is vulnerable to various factors such as periwinkle plant diseases during growth, natural disasters, pandemics, and disruptions of global logistics. Due to the lack of alternatives as well as the tight supply of raw materials, the FDA has listed vinblastine as a shortage drug in 2019−2020. Therefore, alternative and cheap resources are in urgent need for producing such alkaloids to complement a stable supply chain, independent of their natural plant producers.

Synthetic biology has opened the gate for producing natural products that are originally derived from rare medicinal plants or cultivated medicinal plants by fermentation through reprogramming their biosynthetic pathways. Compared with original extraction and purification from medicinal plants or by chemical synthesis, tremendous advantages of the microbial fermentation had been exemplified by engineering *Saccharomyces cerevisiae*, a generally safe strain for the successful manufacture of many plant-derived compounds including antimalarial drugs [4], opioids [5], and cannabinoids [6], etc. The microbial fermentation is a simple process that uses simple renewable substances (carbon sources, nitrogen sources, etc.), and is completely independent of crop factors such as plant diseases and natural disasters. However, microorganisms often lack the genes required for synthesizing plant-derived drugs, especially those with very complex chemical structures. Thus, the genes should be introduced into microorganisms to ensure compatibility and full functionality. Many problems and uncertainties will be encountered during this process. Specifically, how to program dozens of catalytic steps-by-step reactions in a single yeast cell? How to ensure that they are functionally expressed as originally programmed in plant cells? It is often the case that these processes take place in various compartments (cytoplasm, plastid, endoplasmic reticulum, nucleus, and vacuole), and then communicate between different tissues and cell types through transportation. Moreover, other factors, such as maintaining a steady supply of precursors and cofactors throughout the complex pathway, as well as supplying yeast cells with the metabolic flux required for normal cell growth, could also be important.

An article in *Nature* [7] demonstrates clearly a long and complex biosynthetic pathway from yeast natural metabolites geranyl pyrophosphate and tryptophan to vinblastine involving 30 enzymatic steps and one chemical step. A total of 56 genes were edited, including expression of 34 heterologous genes from plants, and optimization of 10 native yeast genes for key precursors. The success of this study was largely due to the discovery of the 31-step biosynthetic pathway of vinblastine, particularly after the last two enzymes were identified in 2018 [8, 9]. In periwinkle plants, the enzymes involved in vinblastine synthesis are distributed in at least five compartments, and transportation of them takes place between different compartments. Based on the substrate and product availability, the authors divided the vinblastine pathway into three distinct modules: the biosynthetic pathway of strictosidine, a common precursor for all natural MIAs, and the biosynthetic pathway of catharanthine and vindoline. Each module was constructed in yeast and tested individually to verify their function. Finally, the yeast strains that incorporated all three modules and optimized module copies were able to produce vindoline and catharanthine from glucose and tryptophan in fed-batch fermentations at titers of 13.2 and 91.4 μg/L, respectively. Unfortunately, there was a failure to express the peroxidase in yeast for the final step of catalyzing the condensation of vindoline and catharanthine to produce vinblastine. To solve this issue, the authors attempted chemical coupling of the purified vindoline and catharanthine from the engineered yeast, finally yielding vinblastine at 23.9 μg/L. Together, the yeast fermentation process that mimics plant biosynthesis, plus an additional chemical condensation, resulted in the successful biochemical synthesis of vinblastine.

Overall, this study successfully synthesized the essential anticancer drug vinblastine via microbial fermentation, which was entirely independent of crop factors. The strategy can be used for the *de novo* synthesis and scaling up of many other complex biosynthetic pathways such as those with high complex structures, or with difficulties in the synthesis or purification using traditional methods. This study certainly opens the door to the possibility of using yeast cells as a versatile chassis to synthesize other MIAs or even analogues that are not naturally occurring. The study provides another strong proof-of-concept of using synthetic biology to generate cheap and renewable substances for the production of drugs that would otherwise derive from plants, thereby relieving drug shortages in the future by utilizing sustainable, economical, farmed-/grown-independent methods of efficient production.
